# BEHACOM - a dataset modelling users’ behaviour in computers

**DOI:** 10.1016/j.dib.2020.105767

**Published:** 2020-05-28

**Authors:** Pedro M. Sánchez Sánchez, José M. Jorquera Valero, Mattia Zago, Alberto Huertas Celdrán, Lorenzo Fernández Maimó, Eduardo López Bernal, Sergio López Bernal, Javier Martínez Valverde, Pantaleone Nespoli, Javier Pastor Galindo, Ángel L. Perales Gómez, Manuel Gil Pérez, Gregorio Martínez Pérez

**Affiliations:** a Department of Information Engineering and Communications, University of Murcia, Murcia 30100 Spain; b Telecommunication Software & Systems Group, Waterford Institute of Technology, Waterford X91 P20H Ireland; c Department of Computer Engineering, University of Murcia, Murcia 30100 Spain

**Keywords:** Behavioural dataset, computer, application statistics, mouse movements, keyboard activity, resource usage

## Abstract

This paper details the methodology and approach conducted to monitor the behaviour of twelve users interacting with their computers for fifty-five consecutive days without preestablished indications or restrictions. The generated dataset, called BEHACOM, contains for each user a set of features that models, in one-minute time windows, the usage of computer resources such as CPU or memory, as well as the activities registered by applications, mouse and keyboard. It has to be stated that the collected data have been treated in a privacy-preserving way during each phase of the collection and analysis. Together with the features and their explanation, we also detail the software used to gather and process the data. Finally, this article describes the data distribution of the BEHACOM dataset.

Specification table

**Table:** 

Subject area	Engineering, Computer Science
More specific subject area	Profiling, Artificial Intelligence
Type of data	CSV files
How data were acquired	Client application for Windows and Linux operating systems
Data format	Raw data
Parameters for data collection	To model the behaviour of each individual with his/her personal computer, we have considered the keyboard, mouse, application usage statistics and resource consumption dimensions
Description of data collection	First, we have gathered the raw data generated by each individual interacting with his/her device. After that, we have aggregated the raw data in time windows of one minute each and have calculated relevant features for each dimension. Finally, we have created vectors of features, have labelled them with the corresponding user identifier and have stored them in the BEHACOM dataset
Data source location	CyberDataLab - Department of Information and Communications Engineering, University of Murcia, Spain
Data accessibility	**Data repository**: BEHACOM [1] Data identification number: 10.17632/cg4br62535.2Direct URL to data: https://data.mendeley.com/datasets/cg4br62535/2**Source code repository**: AuthCode [2] Source code URL: https://github.com/CyberDataLab/AuthCode

## Value of the data

• This dataset covers the existing gap in the literature regarding machine-learning-ready datasets modelling users’ behaviour when they continuously interact with personal computers.
• Academic and scientific community focused on the machine-learning field is the primary recipient of this dataset.
• The application field of this dataset can range from continuous authentication systems, where anomaly detectors and classifiers are trained with the dataset to detect and authenticate users, to users’ profiling applicable to several heterogeneous scenarios.
• The keyboard, mouse, application statistics and resource consumption dimensions make this dataset unique and rich to provide the scientific community with additional and improved data characterisation.

## 1 Data description

**Fig. 1:**
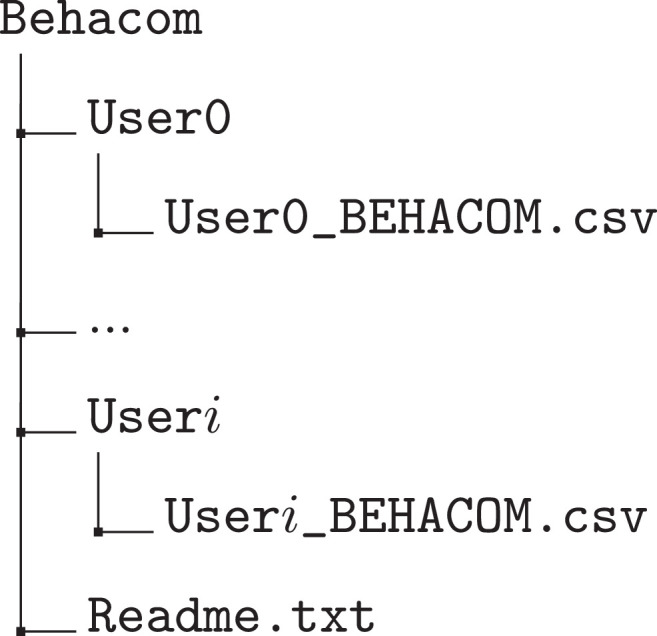
Data repository [1] structure.

**Fig. 2:**
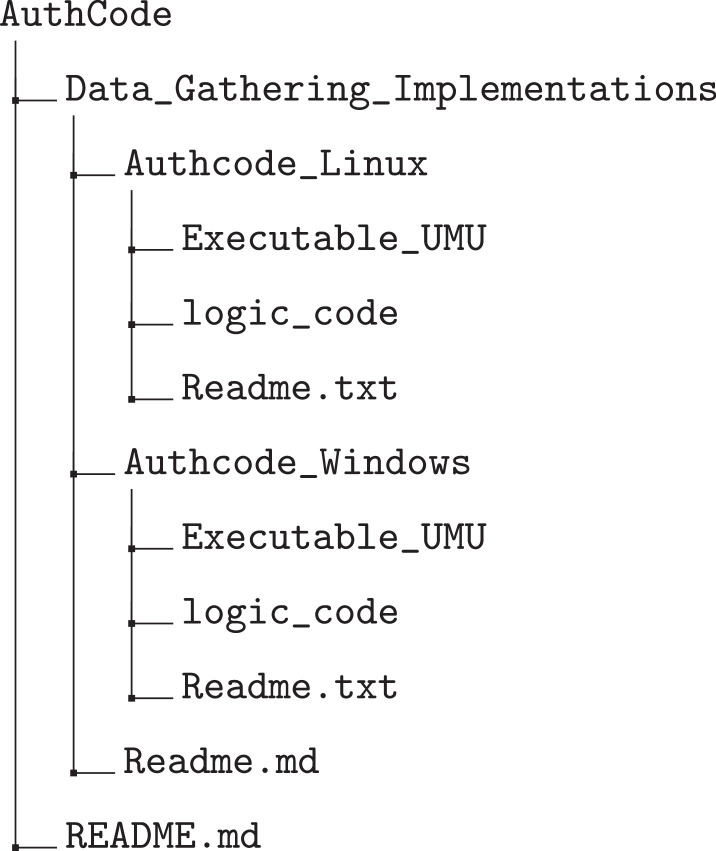
Code repository [2] structure.

**Fig. 3:**
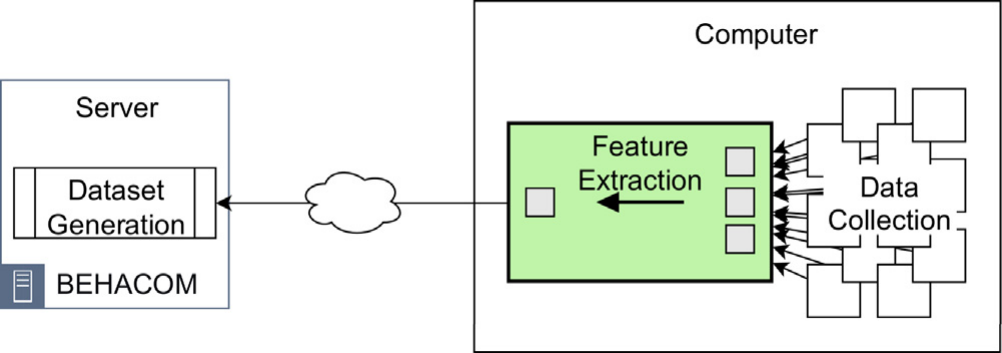
Architecture design.

**Fig. 4:**
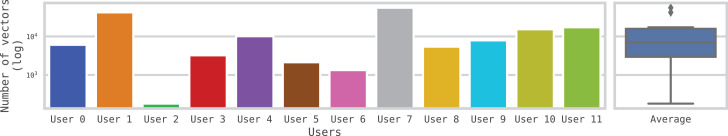
Number of vectors per user

**Fig. 5:**
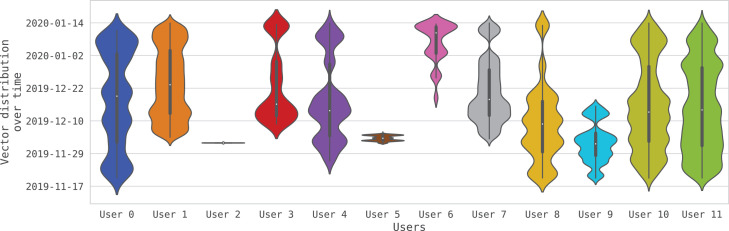
Vectors distributions over time per user

**Fig. 6:**
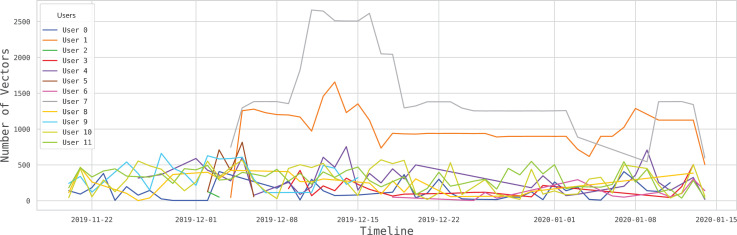
Vector volumes over time per user

**Fig. 7:**
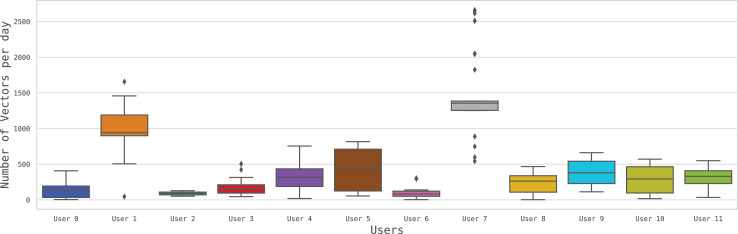
Daily distributions per user

The BEHACOM dataset aims to provide a set of heterogeneous features that models the users’ behaviour when they interact with their personal computers. With that goal in mind, this dataset considers the applications usage statistics, keyboard and mouse actions, and resource consumption of twelve users interacting with their computers for fifty-five days. In this manuscript we provide several tables and figures describing the nature of our work. In terms of figures, Fig. 1 and 2 show data and code description, respectively. Fig. 3 depicts design of the architectural proposed to monitor users and create the dataset. The data characterization of BEHACOM can be seen in Fig. 4, 5, 6 and 7. Regarding tables, the features considered by BEHACOM are described in Table 1, 2, 3, 4, 5, 6.

**Table 1:** Summary of collected features.

Feature Group	Scope	Number	Section
Keyboard	Model the user’s behaviour with the keyboard	11 989	2.1.1
Mouse	Model the events of the mouse	45	2.1.2
App Usage	Model the statistics of the applications usage	7	2.1.3
Resources	Model the computer resource usage	10	2.1.4

**Table 2:** List of separators used to calculate keyboard features

Character separators
-, _, ‘.’, ‘,’, /, &, +, <, space, \t, \n, (, ), =, |, \, #

**Table 3:** Keyboard features description

Feature	Description	Domain
timestamp	Timestamp that indicates when the vector was generated. It contains a numerical value corresponding to the milliseconds elapsed since the UNIX epoch, 1st January 1970.	N
keystroke_counter	Total number of keystrokes generated by the user during the time window.	N
erase_keys_counter	Number of presses of erasing keys. That is, the keystrokes on ‘delete’ and ‘backspace’ keys.	N
erase_keys_percentage	Percentage of erasing keystrokes over the total number of keystrokes.	R
press_press_average_interval	Average time elapsed between two consecutive keystrokes, measured in milliseconds.	R
press_press_stddev_interval	Standard deviation on the time elapsed between two consecutive keystrokes, measured in milliseconds.	R
press_release_average_interval	Average time elapsed between the press and the release of all the keystrokes occurred during the time window, measured in milliseconds.	R
press_release_stddev_interval	Standard deviation of the time elapsed between the press and the release of all the keystrokes occurred during the time window, measured in milliseconds.	R
word_counter	Total number of words typed by the user during the time window. To determine if the user has written a word, alphanumeric keys are used as word characters and character separators list is used as word delimiter characters.	N
word_average_length	Average length of all the words written during the time window.	R
word_stddev_length	Standard deviation of the length of all the words written during the time window.	R
word_length_*N*	Set of features that represents the length histogram of the words written during the time window. *N* ranges from 1 to 11, the words larger than 11 characters are assigned to the 11 box.	N
keystrokes_key_*K_i_*	Set of features that counts the number of keystrokes per key. *K_i_* is the ith key in the list of all possible keys *K*.	N
press_release_average_*K_i_*	Set of features that represents the average time elapsed between the press and the release of each key, measured in milliseconds. Again, *K_i_* is each key in the set *K* of possible keys.	R
digraph_counter_*K_i_K_j_*	Set of features that counters the number of two keys combinations (digraph) introduced by the user. *K* is the list of all the possible keys, and *K_i_K_j_* represents each possible key pair. The features of non-typed digraphs during the time window will have 0 value.	N
digraph_average_time_*K_i_K_j_*	Set of features that represents the average time elapsed to press each possible digraph, measured in milliseconds. Again, *K_i_K_j_* is each possible pair of keys.	R

N: Natural numbers, R: Real numbers

**Table 4:** Mouse features description

Feature	Description	Domain
click_speed_aveage_*N*	Set of features that represents the average time elapsed to complete a click using the mouse (button press-release time). *N* represents each one of the mouse buttons, 0 is left button click, 1 is right button click, 2 is left button double click and 3 is middle button click. These features are measured in milliseconds.	R
click_speed_stddev_*N*	Set of features that represents the standard deviation of the time elapsed to complete a click using the mouse (button press-release time). *N* represents each one of the mouse buttons: 0 is left button single click, 1 is right button click, 2 is left button double click and 3 is middle button click. These features are measured in milliseconds.	R
mouse_action_counter_*N*	Set of features that counters the number of events related to the mouse activity. *N* represents each possible action: 0, is a left button single click, 1 is a right button click, 2 is a left button double click, 3 is a scroll action, 4 is a mouse pointer movement, 5 is a drag or selection action (left press-movement-release), 6 is a middle button click.	N
mouse_position_histogram_*N*	Set of features that counters the number of mouse events occurred in each quadrant of the screen. The screen is organised in 9 quadrants, dividing the screen size into 3 values in width and 3 in height (N∈{1,⋯,9}).	N
mouse_movement_direction_ histogram_*N*	Set of features that represents the number of mouse movement events in each direction, organised in 8 cardinal points based on the movement angles (N∈{1,⋯,8})	N
mouse_movement_length_ histogram_*N*	Set of features that represents the length histogram of the mouse movements occurred during the time window. *N* has 3 possible values, 1: the movement length is less than a third of the screen size, 2: the movement length is between 1/3 and 2/3 of the screen size, 3: the movement length is larger than 2/3 of the screen size.	N
mouse_average_movement_ duration	Average duration of the mouse movements, measured in milliseconds.	R
mouse_average_movement_ speed	Standard deviation of the duration of the mouse movements, measured in milliseconds.	R
mouse_average_movement_ speed_direction_*N*	Set of features that represents the histogram of movement average speeds per direction. N∈{1,⋯,8} like the movement direction histogram.	R

N: Natural numbers, R: Real numbers

**Table 5:** Application usage statistics features description

Feature	Description	Domain
active_apps_average	Average number of applications active during the time window.	R
current_app	Application executable name in foreground when the vector was generated.	S
penultimate_app	Penultimate application executable name in foreground during the time window. If only one application was used during the time window, this application will have ‘-’ as value.	S
changes_between_apps	Number of changes between different foreground applications during the time window.	N
current_app_foreground_time	Number of seconds that the current application has been in foreground during the time window. If no application changes occur, the value of this feature will be 60.	R
current_app_average_processes	Average number of processes that the current application in foreground had active during the time window.	R
current_app_stddev_processes	Standard deviation of the number of processes that the current application in foreground had active during the time window.	R

N: Natural numbers, R: Real numbers, S: Strings

**Table 6:** Resource consumption features description

Feature	Description	Domain
current_app_average_cpu	Average percentage of CPU used by the current application during the time window.	R
current_app_stddev_cpu	Standard deviation of the percentage of CPU used by the current application during the time window.	R
system_average_cpu	Average of the percentage of system CPU capacity used in total during the time window.	R
system_stddev_cpu	Standard deviation of the percentage of system CPU capacity used in total during the time window.	R
current_app_average_mem	Average memory percentage used by the current application during the time window.	R
current_app_stddev_mem	Standard deviation of the percentage of memory used by the current application during the time window.	R
system_average_mem	Average of the percentage of system memory capacity used in total during the time window.	R
system_stddev_mem	Standard deviation of the percentage of system memory capacity used in total during the time window.	R
received_bytes	Number of bytes received through the network interfaces of the device during the time window.	N
sent_bytes	Number of bytes sent using the network interfaces of the device during the time window.	N

N: Natural numbers, R: Real numbers

### 1.1 Data repository

The data repository of BEHACOM is publicly available in Mendeley Data [1]. As it can be seen in Fig. 1, it is organised in the Behacom main folder, which contains a Readme.txt file with the explanation of the dataset features and twelve folders, named User*i* (the value of *i* ranges from 0 to 11). Each folder contains a CSV file, named User*i*_BEHACOM.csv, with a set of feature vectors that group the behaviours of each individual in time windows of one minute each. The CSV files use *iso-8859-1* (*latin-1*) charset due to the special characters present in some users’ data. See Section 2.3 for further details on the content of each CSV file.

Table 1 reports a summary of the collected feature groups that will be discussed in Sections from 2.1.1 to 2.1.4.

### 1.2 Code repository

With the objective of monitoring and storing the behaviour of individuals using personal computers with different operating systems, we have implemented a data collection application for Windows, the most used desktop operating system, and another for Linux distributions based on Debian. The code and implementation details of both applications are available at the UMU CyberDataLab GitHub [2] and belong to the research project of the UMU CyberDataLab named AuthCODE [3]. As observed in Fig. 2, the Data_Gathering_Implementations is the main directory which contains the Authcode_Linux and Authcode_Windows folders. Each folder contains a Readme.txt file, the executable files and the Python source code files implementing the data acquisition functionality for both operating systems. The previous files are organised in two folders, namely the Executable_UMU and the logic_code, providing the executables and the Python source code files, respectively. See Section 2.2 for further details regarding each source code functionality.

## 2 Experimental Design, Materials and Methods

This section explains the details of the scenario where the BEHACOM dataset has been created. It also describes the followed architectural approach and its implementation details to gather the individuals’ behaviour. Finally, it shows the data distribution of each individual’s behaviour contained in BEHACOM.

### 2.1 Scenario description and selected features

The proposed scenario comprises twelve different individuals interacting for fifty-five consecutive days with their personal computers in their own way and without restrictions. The twelve individuals are right-handed male, with ages ranging from 20 to 45 years old. Eight of them use Windows as operating system, three Linux, and one both. Furthermore, ten are Spanish and two Italian. Apart from using their native language, several use English as a working language, which affects the keyboard features considered by the BEHACOM dataset. The first timestamp (UNIX ms) of the dataset is 1574245230186 (Wednesday, 20-Nov-19 10:20:30 UTC) and the last one is 1578995678310 (Tuesday, 14-Jan-20 09:54:38 UTC).

To model the behaviour of each individual with his/her personal computer, we have considered the following four dimensions: keyboard, mouse, application usage statistics and resource consumption. Below we show the list of features considered for each dimension. Before detailing each one of the dimensions and their associated features, it is important to highlight that features have been calculated from raw data grouped in time windows of one-minute to guarantee the privacy of sensitive data and avoid data leakages, especially in the case of keyboard data.

#### 2.1.1 Keyboard

To calculate the set of features modelling the behaviour of each individual with his/her keyboard, we have considered the keys (alphanumeric, control, function and navigation) of Italian and Spanish qwerty keyboards [4]. In addition, we have considered the separator characters detailed in Table 2 to calculate some of the features considered in the BEHACOM dataset, as listed in Table 3.

#### 2.1.2 Mouse

Regarding the activity of each individual with the mouse or trackpad of his/her personal computer, Table 4 describes the features considered in the BEHACOM dataset.

#### 2.1.3 Application Usage Statistics

In terms of application usage statistics, Table 5 outlines both the individual features and the set of them considered in the BEHACOM dataset.

#### 2.1.4 Resource Consumption

Finally, in terms of computer resource consumption, Table 6 describes the set of features that our dataset considers modelling the individuals’ behaviour.

### 2.2 Architectural approach

To generate the BEHACOM dataset, we have designed and implemented an architecture made up of three components: *Data Collection, Feature Extraction* and *Dataset Generation*.

The Data Collection component runs on each personal computer and gathers the raw data generated by each individual interacting with his/her device. After that, the Feature Extraction component aggregates the raw data in time windows of one minute and calculates relevant features for each dimension. Features are grouped in vectors, which are sent periodically to the Dataset Generation component. Finally, this component receives the feature vectors, labels them with the identifier of the proper individual and stores them in the BEHACOM dataset. It has to be remarked that, throughout the proposed analysis, the collected data are treated in a privacy-preserving way because sensitive pieces does not leave the computers. Furthermore, raw data is destroyed once features are collected and sent to the server. Fig. 3 shows the components making up the proposed architecture to create BEHACOM.

#### 2.2.1 Data collection

The Data Collection component continuously monitors the data generated when the user interacts with the keyboard, mouse, applications and resources of his/her personal computer. Below, we detail the raw data considered for each dimension, which is temporally stored in the computer for further processing.

• Keyboard: Timestamp, key press, key release, key and application in foreground.
• Mouse:
  – Mouse movement events: Timestamp, pointer *x* coordinate, pointer *y* coordinate, application in foreground.
  – Mouse click events: Timestamp, pointer *x* coordinate, pointer *y* coordinate, button (left/right/middle), press/release, application in foreground.
  – Mouse scroll events: Timestamp, pointer *x* coordinate, pointer *y* coordinate, movement *x* coordinate, movement *y* coordinate, application in foreground.
• Application and resource usage: Timestamp, number of applications active, name of the current application in foreground, percentage of CPU used by the process in foreground, percentage of CPU used by the application in foreground, total percentage of CPU in use, percentage of memory used by the application in foreground, total percentage of memory in use, bytes received, bytes sent, number of processes of the application in foreground.

To collect and store the previous pieces of data, the Data Collection component has been implemented in Python and the code is based on the following scripts, which are similar for Windows and Linux versions.

• __init__.py. It starts the functionality of collecting behavioural data. Concretely, this script manages the user’s registration/login process, the creation of temporal log files that store raw data until they are sent and deleted, and the execution of the *logger.py* script to initiate the data collection process.
• logger.py. It represents the core of the data collection activity. This script implements the keyboard, mouse and application monitoring logic. In addition, it calls *extract_features.py* each minute to process the raw events and send the derived features to the collecting server.
  – Mouse monitoring. We use the *pynput* [5] library to register movement, click and scroll mouse events.
  – Keyboard monitoring. We utilise the *Keyboard* library [6] to register keyboard events. Despite the fact that *pynput* also supports keyboard event monitoring, it presents an issue in terms of collecting special characters [7], so we use the *Keyboard* library.
  – Application and resource usage monitoring. To obtain the process identifier (pid) of the application in foreground, *xdotool* [8] is used in the Linux implementation and *pywin32* (*win32process* and *win32gui*) [9] in Windows. Process names, CPU, memory and network usage are obtained by the *psutil* [10] library, in both operating systems.

#### 2.2.2 Feature extraction

As the Data Collection component gathers and temporally stores the previous pieces of data, the Feature Extraction component aggregates them in time windows of one minute to calculate vectors comprised of the features indicated in Section 2.1. The aggregation task calculates a set of features that are representative to model the users’ behaviours but without containing user’s sensitive information. In this context, each minute the processing is performed, and the feature vector is sent to our server through a REST API. Using this architectural approach, the application implementing the Data Collection and the Feature Extraction components does not consume excessive storage resources (raw data is deleted after sending each feature vector), and the raw data do not leave the device, reducing the possibility of attacks affecting data leakages and being privacy-preserving.

To achieve the previous functionality, the Feature Extraction component implements the extract_features.py script. It reads the log files of keyboard, mouse and applications, as well as processes them in order to derive the activity features. Raw data related to keyboard and mouse events are read from mouse_keyboard_log_service.txt. Each line of this file contains one event of keyboard and its information, sorted by timestamp. Then, each line is processed to generate the features modelling keyboard and mouse interactions. Raw data about application and resource usage are obtained from apps_log_service.txt. This file is read line by line, extracting the periodic measurements (every 5 seconds) about active applications and resources in use. Then, the features of the application dimensions and resource usage are calculated. Once logs have been processed, all the derived features are grouped in a vector and this vector is sent to our external server utilising a REST API (POST operation).

#### 2.2.3 Dataset generation

The Dataset Generation component receives the feature vectors, it labels them with the user’s identifier and stores the vector in the proper User*i*_BEHACOM.csv file, mentioned in Section 1.2. Finally, before publishing the dataset we have removed the features with constant values for all users. It is important to highlight that vectors have been labelled to ease the task of grouping them in a global and unique dataset. This process is carried out through the following two scripts.

On the one hand, the server.py script launches a web service that exposes a REST API in charge of managing the register and login processes required to receive feature vectors. It utilises Flask [11]. Login and register tasks are managed using GET and POST operations, respectively. When each vector is received, a feature with the user ID is appended in order to identify the vector owner. Then, the labelled vector is stored in the User*i*_BEHACOM.csv file.

On the other hand, the generate_header.py script generates a text file named header.txt which contains all the feature names in the same order that they are in each feature vector, separated with a comma. This file is used as a header when each user’s vector file is read in CSV format.

To conclude the dataset generation, a manual feature preprocessing is performed, removing features with constant values for all users. In this context, the *digraph_counter_KK* and the *digraph_average_time_KK* sets of features, belonging to the keyboard dimension (detailed in Section 2.1), combine all the available keys pairs twice, including some key digraphs never used by individuals. Thus, a significant number of features do not contain relevant information and they are eliminated to reduce dataset size and loading time. With this approach, the initial 24 065 features are reduced to 12 051, the final amount of features that make up the BEHACOM dataset.

### 2.3 Data characterisation

This section aims to show the data distribution of the BEHACOM dataset. In this context, Fig. 4 illustrates the number of feature vectors belonging to each user modelled in BEHACOM. As it can be seen, there is a relevant disequilibrium in terms of number of vectors for the different users, which is highly influenced by the users’ behaviour. In addition, the mean number of vectors per user is about 8 000 (ranging from 5 000 to 15 000 as shown in the average bar), having two users, *User 1* and *User 7* with a number of vectors above 40 000 and 50 000, respectively.

In order to see how the previous vectors are distributed across the time, Fig. 5 depicts, for each user, the normalised distribution of vectors per each day of the fifty-five that the monitoring process has been executed. As shown, *User 0, User 4, User 8, User 10* and *User 11* have activity almost every day of the monitoring period, while the rest of users have periods of inactivity.

In addition to the previous time distributions, Fig. 6 shows the number of vectors generated by each user over time. By looking at the different users, it is noticed that, between the 10th and 18th of December, *User 7* increased his/her activity in a relevant way, jumping from 1 500 vectors to 2 500. The rest of users rarely exceed the threshold of 500 vectors per day.

Finally, Fig. 7 depicts the daily distribution of vectors per user. In this plot, we can see a clear difference between the *Users 1* and *7* and the rest of individuals. They both have the two highest mean numbers of vectors per day (about 900 and 1400, respectively) as well as the two highest standard deviations. For the rest of users, the mean number of vectors ranges from 200 to 400.

## 3 Limitations

The features related to the keyboard activity have been obtained from Spanish and Italian users, with the particularity that some of them also use English as working language. It implies that the use of keyboard features in lexical or language analysis applications is limited to Spanish, Italian or English. In this context, there are special characters like *ñ* or *ç* that does not appear in other languages. Similarly, particular characters and syntax of other languages are not considered in the BEHACOM dataset.

To monitor as many users as possible, we implemented the application able to collect data and extract features for both Windows and Linux operating systems, as indicated in Section 2.2. Since there are some applications not available for one of both operating system, or even the name of the same application could be different in Windows and Linux, it could affect the BEHACOM dataset usage.

Since users’ activity has been monitored when each user freely utilised his/her personal computer, time is a relevant aspect to take into account. In this context, there are periods of time in which the computer is on, but the user is not performing any activity on it. An example of these periods is to keep the computer on during the night but without human intervention. In consequence, these periods will have their corresponding vectors with the keyboard, mouse and application features set to zero, and resource consumption features will have similar values.

Finally, as observed in Section 2.3, another limitation of the BEHACOM dataset is its disequilibrium. The fact that each user has a different behavioural pattern implies that users that are spending more time with their computers will have more vectors than their counterparts.

## CRediT authorship contribution statement

**Pedro M. Sánchez Sánchez:** Conceptualization, Investigation, Validation, Writing - original draft, Software. **José M. Jorquera Valero:** Validation, Writing - original draft. **Mattia Zago:** Data curation, Formal analysis, Visualization. **Alberto Huertas Celdrán:** Investigation, Validation, Writing - original draft. **Lorenzo Fernández Maimó:** Data curation, Writing - review & editing. **Eduardo López Bernal:** Writing - original draft. **Sergio López Bernal:** Data curation, Writing - original draft. **Javier Martínez Valverde:** Writing - original draft. **Pantaleone Nespoli:** Data curation, Writing - review & editing. **Javier Pastor Galindo:** Software, Visualization. **Ángel L. Perales Gómez:** Software, Formal analysis. **Manuel Gil Pérez:** Writing - review & editing, Project administration. **Gregorio Martínez Pérez:** Supervision, Project administration, Funding acquisition.

## Declaration of Competing Interest

The authors declare that they have no known competing financial interests or personal relationships which have, or could be perceived to have, influenced the work reported in this article

## Appendix A Supplementary materials

Supplementary Data S1 Supplementary Raw Research Data. This is open data under the CC BY license http://creativecommons.org/licenses/by/4.0/ Supplementary Data S1
